# Supplementary material for: Global stock structure of the Silky shark (Carcharhinus falciformis, Carcharhinidae) assessed with high-throughput DNA sequencing
Source: PeerJ. 2025 Jul 7;13:e19493. doi: 10.7717/peerj.19493 (PMC12244132; doi:10.7717/peerj.19493)
Supplement: Supplemental Information 2 [file peerj-13-19493-s002.docx]

**Supplemental Material**

**Table S1.** SNP filtering parameters

| AssessPool Filter | Selected for this study | Explanation |
| --- | --- | --- |
| min.pool.number | 2 | Minimum number of pools a SNP need to be present in to be included. Using 2 can quantify small differences between each pairwise comparison. |
| min.quality.score | 20 | Drops SNPs with Phred quality scores lower than this threshold. For this data set 20 was still conservative without over filtering the data. |
| min.depth.threshold | 30 | Drops SNPs with less than 30 coverage. 30 is about the average? pool size so it assumes good coverage of all individuals in the pool as recommended by Schlötterer (2014). |
| max.missing | 3 | Maximum amount of dropped genotypes due to low coverage for a SNP to be included. 3 was selected so SNPs had to be present in at least 1 pool. |
| max.allele.length | 10 | Drops SNPs with an allele length greater than this threshold. 10 drops large indels from the data set. |
| quality.depth.ratio | 0.25 | Drops SNPS with a quality score:depth ratio lower than this threshold which removes low quality, high depth SNPs indicating loci that were likely over-grouped. |
| max.mean.depth.threshold | 1000 | This removes paralogs and multicopy loci, assessPool has graphical output to help select this value based on the dataset. |
